# HitPredict version 4: comprehensive reliability scoring of physical protein–protein interactions from more than 100 species

**DOI:** 10.1093/database/bav117

**Published:** 2015-12-21

**Authors:** Yosvany López, Kenta Nakai, Ashwini Patil

**Affiliations:** ^1^Human Genome Center, The Institute of Medical Science, The University of Tokyo, Tokyo 108-8639, Japan; ^2^Department of Computational Biology, Graduate School of Frontier Sciences, The University of Tokyo, Chiba 277-8561, Japan

## Abstract

HitPredict is a consolidated resource of experimentally identified, physical protein–protein interactions with confidence scores to indicate their reliability. The study of genes and their inter-relationships using methods such as network and pathway analysis requires high quality protein–protein interaction information. Extracting reliable interactions from most of the existing databases is challenging because they either contain only a subset of the available interactions, or a mixture of physical, genetic and predicted interactions. Automated integration of interactions is further complicated by varying levels of accuracy of database content and lack of adherence to standard formats. To address these issues, the latest version of HitPredict provides a manually curated dataset of 398 696 physical associations between 70 808 proteins from 105 species. Manual confirmation was used to resolve all issues encountered during data integration. For improved reliability assessment, this version combines a new score derived from the experimental information of the interactions with the original score based on the features of the interacting proteins. The combined interaction score performs better than either of the individual scores in HitPredict as well as the reliability score of another similar database. HitPredict provides a web interface to search proteins and visualize their interactions, and the data can be downloaded for offline analysis. Data usability has been enhanced by mapping protein identifiers across multiple reference databases. Thus, the latest version of HitPredict provides a significantly larger, more reliable and usable dataset of protein–protein interactions from several species for the study of gene groups.

**Database URL**: http://hintdb.hgc.jp/htp

## Introduction

Knowledge of protein–protein interactions is essential for the understanding of cellular pathways and their functions. Network analysis and pathway prediction methods are commonly used to study groups of genes and predict their functional associations. However, these methods require interaction data of high quality, i.e. reliability, in order to provide meaningful results. The reliability of an interaction indicates the probability that two proteins bind to each other, or are functionally associated, *in vivo*. This probability varies considerably depending on whether the association is direct or indirect. In direct interactions, two proteins are known to physically bind to each other. On the other hand, indirect interactions may be genetic, predicted on the basis of homology and other genomic features, or functional associations between co-expressed genes. Of the two types, direct (or physical) interactions that have been experimentally identified are considered to be a better indicator of physical binding of proteins *in vivo*, and hence more reliable. For physical interactions, reliability also varies considerably depending on the size of the experiment and the method used to identify them. Interactions identified in high-throughput experiments are more likely to be spurious than those from small-scale experiments (1). Thus, physical protein–protein interactions, though of higher quality than genetic or predicted ones, still need to be assessed for reliability.

Several databases provide access to various types of interactions, either as primary sources (2–6) or as consolidated resources (7–12). Some of these databases contain physical interactions (4, 12) while others also include genetic ones (2, 3, 5). A few also include interactions between proteins and nucleic acids as well as predicted associations (7–9) and are species-specific (4, 9, 11, 13). Others automatically integrate interactions from multiple source databases (12). Many of these resources calculate reliability scores for interactions primarily based on the details of the experimental method (2, 5, 7, 12). However, these scores do not take the properties of the interacting proteins into account. Despite the large number of choices available, it is still challenging to extract a reliable set of physical protein–protein interactions from these databases. This is because they either do not contain all known interactions, or contain a mixture of direct and indirect interactions, or they are limited to only one, or a few, species. Further, integration of interactions from multiple databases requires accurate annotations for the interactions such as valid protein identifiers and standardized terms for experimental description. The MIntAct project has recently emerged as an international effort to standardize curation and retrieval of interactions from source databases (2). The Human Proteome Organization Proteomics Standards Initiative-Molecular Interactions (HUPO PSI-MI) consortium has specified well-defined standards for the representation of interaction information and substantial effort has been made by databases to adhere to these standards (14, 15). However, the large amount of data to be standardized and the differences in levels of compliance across databases result in some discrepancies in the data and incorrect annotations. Specifically, all protein identifiers are not always included, protein sequences are often absent and co-complex interactions are directly provided in expanded binary format. In experimental descriptions, one Pubmed ID is often associated with distinct interaction type and experimental method terms in different databases, requiring extensive checking and standardization before integration.

The HitPredict database (http://hintdb.hgc.jp/htp/) addresses these issues by providing a consolidated resource of scored, physical protein–protein interactions from multiple species with extensive manual curation. Interactions, most of which are identified *in vitro*, are scored to predict their probability of occurring *in vivo*. HitPredict was one of the first databases to introduce scoring of protein interactions in 2005 with a unique interaction-scoring algorithm based on the genomic features of interacting proteins (16, 17). In the latest version of HitPredict, we provide a comprehensive reliability score calculated from experimental information and features of interacting proteins, along with improvements in data coverage and accessibility.

## Updates in version 4

Table 1 shows a list of improvements in the latest version of HitPredict.

**Table 1.** Improvements in HitPredict version 4 over version 3

Property	HitPredict version 3	HitPredict version 4
Data sources	3	5
	(IntAct, BioGRID, HPRD)	(IntAct, BioGRID, HPRD, DIP, MINT)
Data coverage	9 species	105 species
	50 200 proteins	70 808 proteins
	245 409 interactions	398 696 interactions
Scoring schema	Annotation-based	Annotation-based
		Method-based
		Combined
Score coverage	Interactions from high-throughput experiments	All interactions
Manual curation	No	Yes
Data visualization	Static network layout	Flexible network layout
Reference mapping	None	UniProt IDs mapped to Entrez and Ensembl IDs
Data download	Entire dataset only	Entire dataset or for a particular protein

### Database content and integration

**Figure 1:**
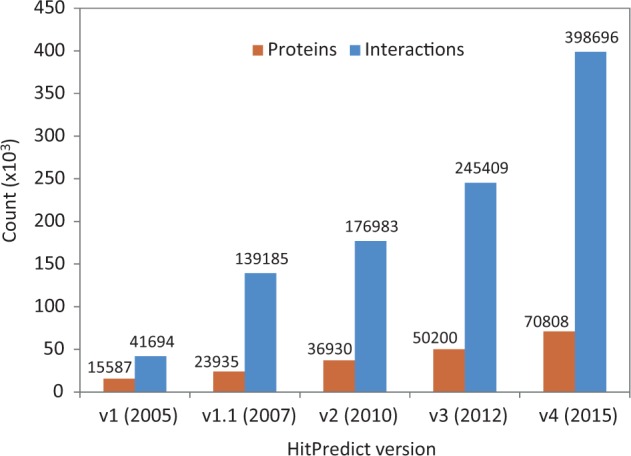
HitPredict database content in all updates from 2005 to 2015.

**Figure 2:**
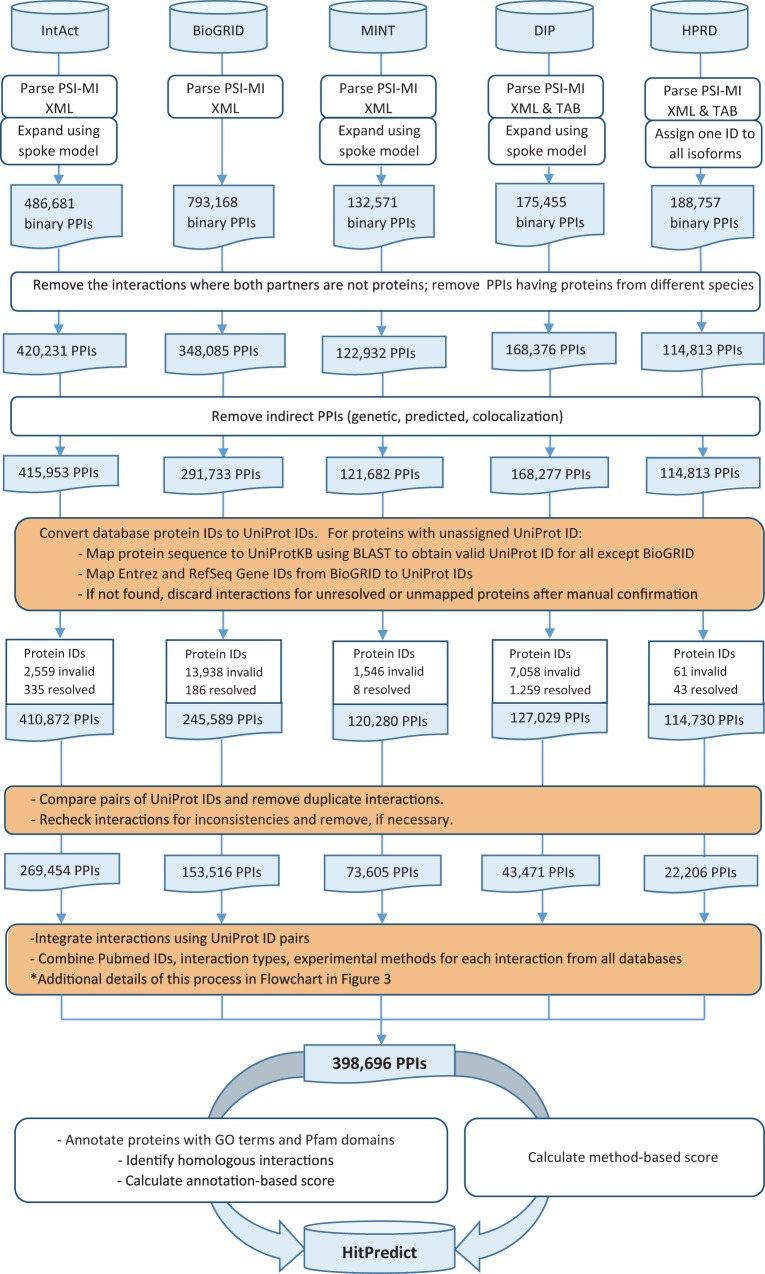
HitPredict interaction data assembly and curation (orange boxes indicate manual curation). PPIs: protein-protein interactions.

Version 4 of HitPredict contains 398 696 interactions among 70 808 proteins from 105 species. The number of proteins and interactions in HitPredict has grown significantly over the last 10 years (Figure 1). The interactions were taken from five source databases (from March 2015). In addition to the interactions from IntAct (2), BioGRID (3) and HPRD (4), the current version also includes those from DIP (6) and MINT (5). Figure 2 shows the detailed methodology used to populate HitPredict with the interaction counts obtained in each step of the process. For all the databases, PSI-MI XML files were processed. In the case of HPRD (4) and DIP (6), tab-delimited files containing binary interactions were also analysed since the bait and prey proteins within protein complexes were not always clearly indicated. Binary interactions identified by methods like yeast two-hybrid were directly taken from the source databases. Protein complexes in IntAct, MINT and DIP were converted to binary interactions using the ‘spoke’ model where each prey protein is assumed to bind to the bait protein. Multiple bait proteins, where present, were assumed to bind to each other. Complexes with no indicated bait proteins were not considered. All interactions in BioGRID were provided in binary format and taken as is. Following the conversion to binary form, interactions where both participants were not proteins, or belonged to different species were removed. Indirect interactions such as genetic, predicted and those based on colocalization were also discarded.

Subsequently, database protein IDs were converted to UniProt IDs. Proteins with unassigned UniProt IDs were remapped to valid IDs by aligning their sequence to that of proteins in UniProtKB (18) using BLAST (99% identity). Entrez and RefSeq Gene IDs, in the case of BioGRID, were mapped to UniProt IDs. In cases where the old UniProt IDs did not map to other valid ones in UniProtKB, the interactions were excluded after manual confirmation. Pairs of UniProt IDs were compared and duplicated interactions were also removed. The combined interactions were finally rechecked for inconsistencies in protein annotations and removed, if necessary. The process of assigning valid UniProt IDs to proteins was challenging because all source databases did not use a common identifier for interacting proteins. While most databases assigned UniProt IDs to proteins, in some cases like BioGRID, Ensembl or Entrez IDs were provided. In cases where UniProt IDs were provided, a significant number were either invalid or obsolete. Additionally, protein sequences were absent from the PSI-MI XML files and were separately retrieved from multiple sources depending on the protein identifier used.

HitPredict uses HUPO PSI-MI defined controlled vocabulary to assign interaction type and experimental method descriptions (14). Only physical interactions indicated by the interaction type terms “association”, “physical association” or “direct interaction” were included. Interactions with “experimental interaction detection” methods other than “genetic interference” were included. Non-standard and obsolete term descriptions no longer supported by the PSI-MI controlled vocabulary were manually removed or remapped to new terms.

**Figure 3:**
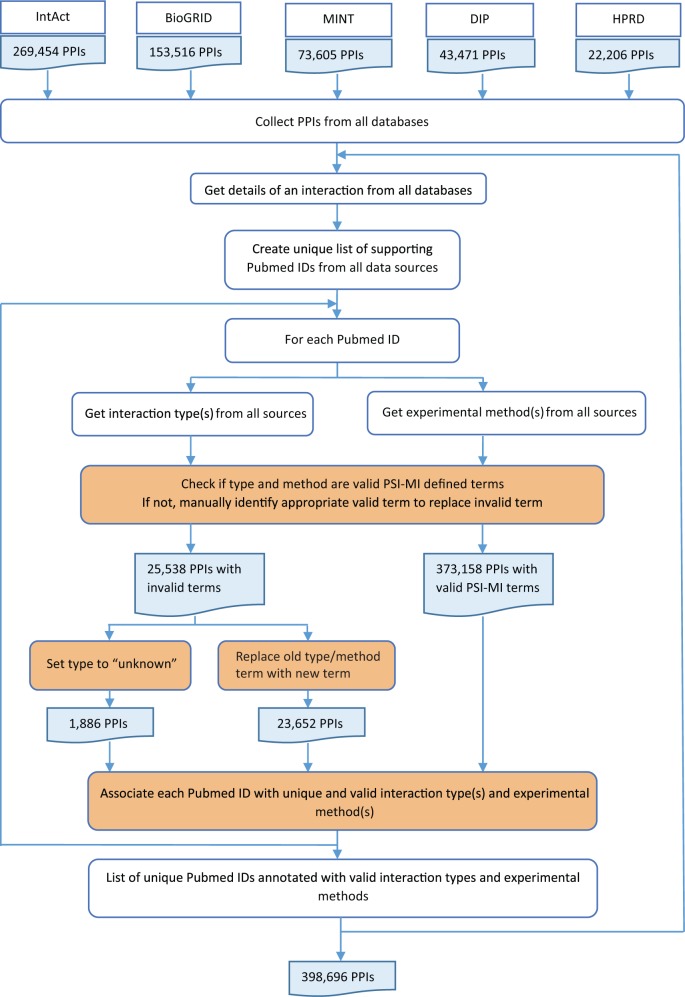
HitPredict experimental information integration and curation. This flowchart shows the process used to combine experimental information from all the source databases for all interactions (orange boxes indicate manual curation). PPIs: protein-protein interactions.

Pubmed IDs, interaction types and experimental methods were combined for each interaction from all source databases (Figure 3). During integration, experimental evidence from all databases was collected for each interaction and a unique list of all supporting Pubmed IDs was created. For each Pubmed ID, the interaction type and experimental method descriptions were obtained from all five source databases. Invalid type and method terms were replaced with valid PSI-MI defined terms after manual confirmation. Invalid terms for which valid replacements could not be found were denoted as ‘unknown’. Each Pubmed ID was associated with unique and valid interaction types and experimental methods as provided by the source databases. This was critical for calculating an accurate score based on experimental information. Despite the standards prescribed and adhered to by the source databases, extensive manual curation was required to remove inconsistent interaction type or experimental method descriptions for the same Pubmed ID across multiple databases.

**Figure 4:**
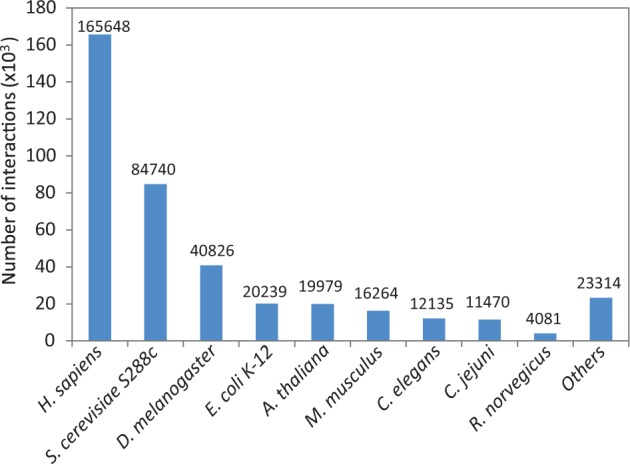
Distribution of physical protein–protein interactions in HitPredict by species.

Species with at least 10 interactions within the combined unique set of protein–protein interactions were added to HitPredict. This resulted in interactions from 105 species (Supplementary Table S1), a significant increase over the previous nine model organisms (Figure 4). With these improvements in data collection, the latest version of HitPredict provides an extended and extensively curated dataset of high-quality protein–protein interactions.

We compared the contents of HitPredict with those of another similar database, mentha. mentha is an interaction database that automatically integrates interactions from The International Molecular Exchange Consortium (IMEx) (15) data sources and provides reliability scoring based on the number of experiments supporting an interaction (12). mentha relies on curation provided by source databases and therefore does not provide additional curation during data integration. A comparison of the interactions from mentha (as of 24^th^ August 2015) with those in HitPredict shows 91% overlap with HitPredict having fewer interactions than mentha (Supplementary Figure S1). However, of the 500 092 interactions in mentha (as of 14^th^ September 2015), 33 199 are among proteins from different species while 479 proteins involved in 1 419 interactions had invalid or obsolete UniProt IDs. On the other hand, interactions among different protein pairs or invalid UniProt IDs have been systematically removed from HitPredict through manual curation resulting in a smaller set of high-quality interactions.

### Interaction scoring

**Figure 5:**
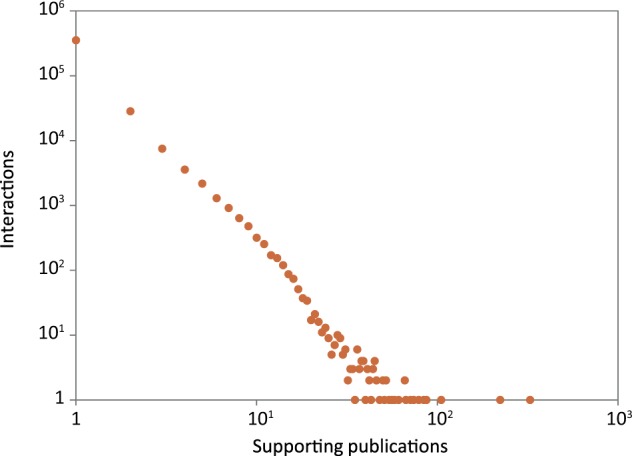
Number of publications supporting the protein–protein interactions in HitPredict.

Reliability scores are useful for assessing the quality of interactions, helping to identify the potentially false or spurious ones. 88% (352 387) of the interactions in HitPredict were supported by a single publication (Figure 5). Additionally, 74% (296 452) were obtained from large-scale experiments identifying >100 associations, and hence considered high throughput (16). Reliability scores are essential to check the quality of these types of interactions, which form the bulk of the data in HitPredict. Various methods have been used to calculate reliability scores. The most prevalent method makes use of experimental information (2, 5, 10, 12), though homology is also used (7). In version 4, HitPredict combines the following two scores derived from complementary information about the experimental method and the binding proteins into a single interaction score.

#### Method-based score

The method-based score considers the experimental details of the interactions. Several databases use this type of scoring to identify high-confidence interactions (2, 5, 12). This score has been introduced into HitPredict version 4 to assess interactions between proteins that lack functional annotations. It was calculated according to the guidelines of the MIScore scoring system specified by the HUPO PSI-MI consortium (19). The MIScore was computed based on the following scores:

1. Interaction detection method score: This score was calculated based on the different types of methods and the frequency with which they were used to identify the interaction. Detection methods used for scoring include ‘biophysical’, ‘protein complementation assay’, ‘biochemical’, ‘post-transcriptional interference’ and ‘imaging technique’. All submethods included in each of the five categories are treated independently and assigned the same score as the parent method. In cases where a valid interaction detection method was not specified by the source database, the interactions are assigned to ‘unknown’ method and consequently given a poor score. Default scores given to each method by the HUPO PSI-MI consortium were used to calculate the final score (19).
2. Interaction type score: This score was calculated based on whether the interaction was physical, genetic or predicted. Since only physical interactions are present in HitPredict, the interaction type was either ‘direct interaction’, ‘physical association’ or ‘association’. This score also takes into account the number of publications, or experiments, that detected the interaction of this particular type. Default scores specified by the HUPO PSI-MI consortium were used (19).
3. Publication score: This score was calculated based on the distinct number of experiments in which the interaction was identified. It varies from 0 to 1 with interactions identified by seven or more publications having the highest value. This score was calculated as specified by the MIScore (19).

The average of these three scores was taken as the method-based score and varies from 0 to 1 (19). The detection method and type scores take into account the number of times an interaction was identified with a specific method or type. This can result in inflation of the score due to the same interaction method or type being obtained from multiple databases. To prevent this, each Pubmed ID supporting an interaction was first associated with unique experimental methods and interaction types as provided by the source database. Each interaction was then associated with a unique list of Pubmed IDs from multiple databases. A list of interaction methods and types was computed for each interaction using the unique list of Pubmed IDs. This list of methods and types was used to compute the final method-based score (Figure 3). An optimal score cutoff of 0.485 has been suggested for the method-based score to identify high-confidence interactions (19).

#### Annotation-based score

The annotation-based score is the original score that has been provided in HitPredict since its inception in 2005 (17). Interactions were assigned this score based on the presence of the following three features:

1. The proteins contain Pfam (20) domains that have been observed to bind in three-dimensional structures in protein complexes (21). This feature assesses the probability of the interaction occurring based on the structural features of the binding proteins.
2. The proteins share at least one Gene Ontology term (22). This feature identifies proteins that share functional associations since proteins with similar functions are more likely to interact.
3. An interaction between the homologs of the two proteins exists in the same or another species. This information is obtained from the HINTdb database (23) wherein homologous interactions are identified using PSIBlast with five iterations and an e-value threshold of 10^−^^8^.

Of the three features, the presence of interacting Pfam domains has been shown to be the best discriminant of true interactions. Interactions supported by all three features have the highest reliability. The ability of each feature to predict the reliability of an interaction was calculated as a likelihood ratio. Likelihood ratios were combined using naïve Bayesian networks to provide a reliability score. A likelihood ratio greater than 1 is an indicator of a high-confidence interaction. Low-confidence interactions without support from any of the above features have a likelihood ratio of 0.163. The likelihood ratio varies with the number of features supporting an interaction and was converted to an annotation score between 0 and 1 (Supplementary Table S2). An annotation score greater than 0.5 corresponds to a likelihood ratio greater than 1 indicating a high-confidence interaction.

#### Combined interaction score

A combined score between 0 and 1 denoting the overall reliability of the interaction was calculated as the geometric mean of the annotation-based and method-based scores. Interactions with a total score greater than 0.281, corresponding to an annotation-based score greater than or equal to 0.5, or a method-based score greater than or equal to 0.485, were considered to be of high confidence.

Earlier versions of HitPredict provided confidence scores for only a subset of interactions that were obtained from high-throughput experiments or expanded from co-immunoprecipitated complexes. Those from small-scale experiments were assigned a high score by default since it was assumed that they were reliable. From version 4, confidence scores have been assigned to all interactions irrespective of the size of the experiment they are identified in.

### Evaluation of HitPredict reliability scores

The annotation, method and combined interaction scores from HitPredict were evaluated for their ability to identify true interactions and compared with the MINT score used by mentha (12). Gold standard positive and negative sets were prepared as follows:

#### Positive set

Yeast interactions in HitPredict and mentha that were supported by at least one small-scale experiment were included. An experiment with less than or equal to 100 associations was considered to be small-scale based on the observation that such interactions have better support from multiple evidences (16).

#### Negative set

Yeast interactions in HitPredict and mentha that were supported only by high-throughput experiments and where the interacting proteins were localized in different cellular compartments (24) were included. An experiment reported with greater than 100 interactions was considered to be high-throughput.

**Figure 6:**
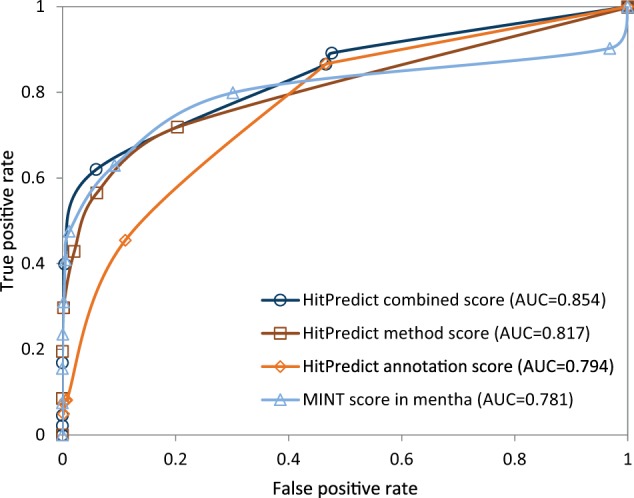
Evaluation and comparison of the HitPredict annotation, method and combined interaction scores with the MINT score in mentha.

The negative set contained 2 160 interactions. Therefore, the same number of interactions was randomly selected from the positive set. For various score thresholds, the number of predicted true positive and false positive interactions were identified and used to plot the Receiver Operating Characteristic (ROC) curve for each score (Figure 6). The area under the ROC curve (AUC) was computed to determine the performance of each score. The results show that the HitPredict combined score (AUC  =  0.854), which is a combination of the annotation score (AUC  =  0.794) and the method score (AUC  =  0.817), performs better than either of the scores individually. All HitPredict scores perform better than the MINT score used by mentha (AUC  =  0.781). Though the MINT score performs slightly better than the HitPredict scores at lower false positive rates, the performance of the HitPredict scores improves at higher sensitivity. Thus, a combination of features and evidences is a more accurate indicator of reliability and combining the two scoring schemes has significantly increased the coverage of high-confidence interactions within HitPredict.

### User interface

The main purpose of the HitPredict user interface is to allow users to search for physical interactions of proteins and identify the ones that are of high confidence. The user can enter a search keyword or a UniProt, Entrez or Ensembl identifier to search for a protein. For each protein, HitPredict displays its interactions along with reliability scores. Interaction networks are made using Cytoscape.js (25) and show 15 binding partners with the highest scores. The graph is dynamically generated with clickable nodes and edges. Edge colors indicate interaction reliability with darker edges denoting higher scores and greater reliability. Clicking on a protein in the networks displays the list of interactions for that protein. Clicking on an edge joining two proteins displays the details of the interaction. The interactions page displays information about the experimental methods used to calculate the method-based score and the features of the interacting proteins from which the annotation-based score was determined. Additional information such as the source database, whether the interaction is identified in a small-scale experiment and whether it is of high confidence is also shown. The user can also come to the interaction page by clicking an interaction ID from the table.

The increased data coverage and the new score can be demonstrated through the interactions of a new species added to HitPredict—the rice plant (*Oryza sativa Japonica Group*) (Supplementary Figure S2 A and B). Searching for the protein MADS6, a MADS-box transcription factor, and going to its interaction page, the user can view several interactions from both small-scale and high-throughput experiments. While the method score for many of these interactions is below the threshold for high confidence, the annotation score is very high and indicates that these interactions are indeed high confidence. Clicking on one of the interactions, for instance that of MADS6 with MAD57 (Interaction ID: 255680), shows the reason for the high annotation score of this interaction. This interaction has several homologous interactions in other plant species (*Arabidopsis thaliana*) along with support from common Gene Ontology terms and interacting Pfam domains. The utility of the method score is shown by the interactions of an uncharacterized protein, O25828, from the bacterium *Helicobacter pylori* (Supplementary Figure S2 C and D). The interaction of O25828 with the protein DNAA has a poor annotation score because of the lack of annotations for the protein O25828. However, this interaction has a very high method score because it has been observed in four separate experiments, thus making it high confidence. The details of the experimental evidence in support of this interaction can be seen on the interaction details page. Thus, the method-based score and the annotation-based score together take into account various aspects of the interaction for the assessment of its reliability.

All the interactions in HitPredict are available for download in tabular and PSI-MI format. The user can download the confidence scores for interactions of a particular protein or species. Mapping of UniProt IDs to Entrez and Ensembl IDs is provided in the downloadable files. These improvements make the latest version of HitPredict easier to access and integrate with other analysis tools.

## Discussion

The focus on direct protein–protein interactions from multiple model organisms, the unique feature-based scoring scheme and the ease of access were the important features of HitPredict in the earlier version (16). The latest version improves HitPredict by increasing the number of species for which interactions are available. A new score combines the previous annotation score and an additional method-based score, which helps in the assessment of interactions of proteins that have insufficient functional annotations. The mapping of protein identifiers across multiple databases has made the dataset easier to use. Manual verification of incorrectly annotated interactions has further increased the overall quality of the data in HitPredict. Extensive efforts have been put into creating a clean and accurately annotated and scored interaction dataset. Despite the availability of standardized formats and terms, this update of HitPredict shows that integration of interaction datasets to obtain a unique set of high-quality interactions requires considerable manual effort. The scoring scheme provided by HitPredict also performs better than the standard MIScore (method score) (19) and the MINT score in mentha (12).

Since its introduction in 2005, HitPredict has been continually improved and updated. UniProt IDs are periodically updated every six months. Future versions will continue to increase data coverage and improve data quality while adding functionality to enhance the usability of the database.

## Supplementary Data

Supplementary data are available at *Database* Online.

Supplementary Data
