# Supplementary material for: HitPredict version 4: comprehensive reliability scoring of physical protein–protein interactions from more than 100 species
Source: Database (Oxford). 2015 Dec 21;2015:bav117. doi: 10.1093/database/bav117 (PMC4691340; doi:10.1093/database/bav117)
Supplement: Supplementary Data [file supp_2015_bav117_index.html]

HitPredict version 4: comprehensive reliability scoring of physical protein–protein interactions from more than 100 species — Supplementary Data 

# HitPredict version 4: comprehensive reliability scoring of physical protein–protein interactions from more than 100 species

## Supplementary Data

files

- Supplementary Data - pdf file
