# Supplementary material for: HitPredict version 4: comprehensive reliability scoring of physical protein–protein interactions from more than 100 species
Source: Database (Oxford). 2015 Dec 21;2015:bav117. doi: 10.1093/database/bav117 (PMC4691340; doi:10.1093/database/bav117)

SUPPLEMENTARY INFORMATION

Table S1. Proteins and interactions from 105 species included in HitPredict.

Taxonomy Id	Species	Proteins	Interactions
9606	*Homo sapiens*	17232	165648
559292	*Saccharomyces cerevisiae S288c*	6019	84740
7227	*Drosophila melanogaster*	10077	40826
83333	*Escherichia coli K-12*	3351	20239
3702	*Arabidopsis thaliana*	7121	19979
10090	*Mus musculus*	6678	16264
6239	*Caenorhabditis elegans*	5011	12135
192222	*Campylobacter jejuni subsp. jejuni*	1296	11470
10116	*Rattus norvegicus*	2399	4081
243276	*Treponema pallidum subsp. pallidum str. Nichols*	710	3542
284812	*Schizosaccharomyces pombe 972h-*	1533	3481
1111708	*Synechocystis sp. PCC 6803 substr. Kazusa*	1909	3326
85962	*Helicobacter pylori 26695*	982	2327
36329	*Plasmodium falciparum 3D7*	1145	2266
224308	*Bacillus subtilis subsp. subtilis str. 168*	684	1535
83334	*Escherichia coli O157:H7*	206	697
272634	*Mycoplasma pneumoniae M129*	384	541
7955	*Danio rerio*	295	385
246197	*Myxococcus xanthus DK 1622*	78	378
10366	*Murid herpesvirus 1*	105	363
341980	*Human herpesvirus 3 strain Oka vaccine*	68	321
9031	*Gallus gallus*	299	321
8355	*Xenopus laevis*	276	310
272951	*Rickettsia sibirica 246*	160	284
39947	*Oryza sativa Japonica Group*	212	230
9913	*Bos taurus*	216	197
170187	*Streptococcus pneumoniae TIGR4*	65	156
69014	*Thermococcus kodakarensis KOD1*	129	127
10298	*Human herpesvirus 1*	48	114
10710	*Enterobacteria phage lambda*	48	98
70601	*Pyrococcus horikoshii OT3*	110	98
562	*Escherichia coli*	94	92
83332	*Mycobacterium tuberculosis H37Rv*	82	88
18101	*Gerbera hybrid cultivar*	14	87
4102	*Petunia x hybrida*	23	78
44689	*Dictyostelium discoideum*	84	77
99287	*Salmonella enterica subsp. enterica serovar Typhimurium str. LT2*	74	69
10335	*Human herpesvirus 3*	29	65
3055	*Chlamydomonas reinhardtii*	48	65
37296	*Human herpesvirus 8*	26	65
10376	*Human herpesvirus 4*	49	64
435895	*Human herpesvirus 8 type M*	34	61
208964	*Pseudomonas aeruginosa PAO1*	66	57
10299	*Herpes simplex virus (type 1 / strain 17)*	39	49
10254	*Vaccinia virus WR*	49	48
237561	*Candida albicans SC5314*	52	47
3888	*Pisum sativum*	32	43
10377	*Human herpesvirus 4 (strain B95-8)*	34	43
211044	*Influenza A virus (A/Puerto Rico/8/1934(H1N1))*	10	38
9986	*Oryctolagus cuniculus*	43	36

4513	*Hordeum vulgare*	40	35
185431	*Trypanosoma brucei brucei TREU927*	37	34
243274	*Thermotoga maritima MSB8*	36	31
176299	*Agrobacterium fabrum str. C58*	25	30
190650	*Caulobacter crescentus CB15*	30	29
623	*Shigella flexneri*	29	28
4577	*Zea mays*	25	28
235443	*Cryptococcus neoformans var. grubii H99*	18	28
10760	*Enterobacteria phage T7*	26	27
574521	*Escherichia coli O127:H6 str. E2348/69*	23	26
367110	*Neurospora crassa OR74A*	23	26
210	*Helicobacter pylori*	20	24
273057	*Sulfolobus solfataricus P2*	30	24
630	*Yersinia enterocolitica*	21	24
243232	*Methanocaldococcus jannaschii DSM 2661*	29	24
33708	*Murid herpesvirus 4*	26	24
199310	*Escherichia coli CFT073*	22	23
1140	*Synechococcus elongatus PCC 7942*	16	22
300852	*Thermus thermophilus HB8*	30	22
4081	*Solanum lycopersicum*	23	22
9615	*Canis lupus familiaris*	27	21
187420	*Methanothermobacter thermautotrophicus str. Delta H*	29	21
9823	*Sus scrofa*	31	20
227321	*Aspergillus nidulans FGSC A4*	11	20
186497	*Pyrococcus furiosus DSM 3638*	26	19
197221	*Thermosynechococcus elongatus BP-1*	19	19
3562	*Spinacia oleracea*	24	19
5691	*Trypanosoma brucei*	12	18
353153	*Trypanosoma cruzi strain CL Brener*	18	18
103690	*Nostoc sp. PCC 7120*	20	18
243277	*Vibrio cholerae O1 biovar El Tor str. N16961*	19	17
282458	*Staphylococcus aureus subsp. aureus MRSA252*	12	17
10747	*Streptococcus phage Cp-1*	15	17
83558	*Chlamydia pneumoniae*	9	16
100226	*Streptomyces coelicolor A3(2)*	18	16
303	*Pseudomonas putida*	18	16
4097	*Nicotiana tabacum*	20	15
274	*Thermus thermophilus*	15	14
59241	*Streptococcus phage Dp-1*	16	14
224324	*Aquifex aeolicus VF5*	15	13
759272	*Chaetomium thermophilum var. thermophilum DSM 1495*	16	13
1422	*Geobacillus stearothermophilus*	15	12
246196	*Mycobacterium smegmatis str. MC2 155*	12	12
267377	*Methanococcus maripaludis S2*	13	12
272943	*Rhodobacter sphaeroides 2.4.1*	15	12
7719	*Ciona intestinalis*	12	11
284590	*Kluyveromyces lactis NRRL Y-1140*	12	11
82830	*Epstein-barr virus strain ag876*	8	11
85963	*Helicobacter pylori J99*	14	11
224325	*Archaeoglobus fulgidus DSM 4304*	14	11
354	*Azotobacter vinelandii*	13	10
1083	*Phaeospirillum molischianum*	5	10
226186	*Bacteroides thetaiotaomicron VPI-5482*	11	10
272844	*Pyrococcus abyssi GE5*	10	10
285224	*Chaetomium thermophilum var. thermophilum*	7	10

Table S2. Conversion of likelihood ratio to annotation score between 0 and 1. A high likelihood ratio indicates support from a greater number of features.

**Likelihood ratio***	**Supporting feature#**	**Annotation score+**	**Interaction Confidence**
0.163	None	0.163	Low
2.575	h	0.5	High
3.370	g	0.55	High
8.678	g+h	0.6	High
19.595	d	0.7	High
50.457	d+h	0.8	High
66.035	d+g	0.9	High
170.041	d+g+h	1.0	High

* Likelihood ratio is calculated for all combinations of features supporting an interaction (Patil and Nakamura, BMC Bioinformatics, 2005).

# d: interacting proteins have Pfam domains that are observed to interact in 3D structures; g: interacting proteins share a common Gene Ontology term; h: interaction has homologs in one or more species.

+ Likelihood ratios are mapped to an annotation score between 0 and 1. The conversion of the likelihood ratio to the annotation score does not affect the ROC curve shown in Figure 6.

Figure S1. Data coverage in HitPredict and mentha interactions.

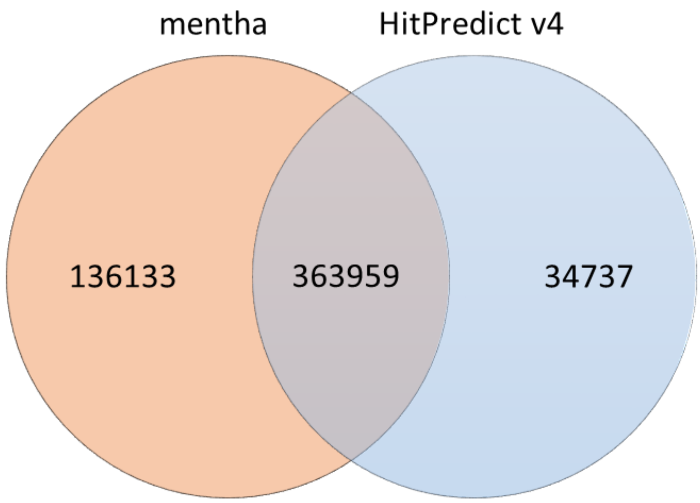

<u>Figure S2.</u> Web interface of the HitPredict database. A) List of interactions for the protein MADS6 from rice (*Oryza sativa Japonica Group*) along with the interaction network and calculated reliability scores. Network nodes and edges are clickable and take the user to the corresponding proteins or interactions page. Edge color denotes interaction reliability. Details of the interaction highlighted in red are shown in (B). This interaction has a poor method score but a very high annotation score, making it high confidence. B) Details of the interaction between MADS6 and MAD57 showing information about the experimental method and the genomic features of the proteins, along with homologous interactions from *A. thaliana* used to calculate the reliability scores. C) List of interactions of an uncharacterized protein O25828 from the bacterium *Helicobacter pylori*. The details of the interaction highlighted in red with the protein DNAA are shown in (D). This interaction has a poor annotation score because one of the proteins is uncharacterized. However, it is supported by four publications and therefore, has a high method score, making it high confidence. D) The details of the experimental support for the interaction between O25828 and DNAA from *Helicobacter pylori*.

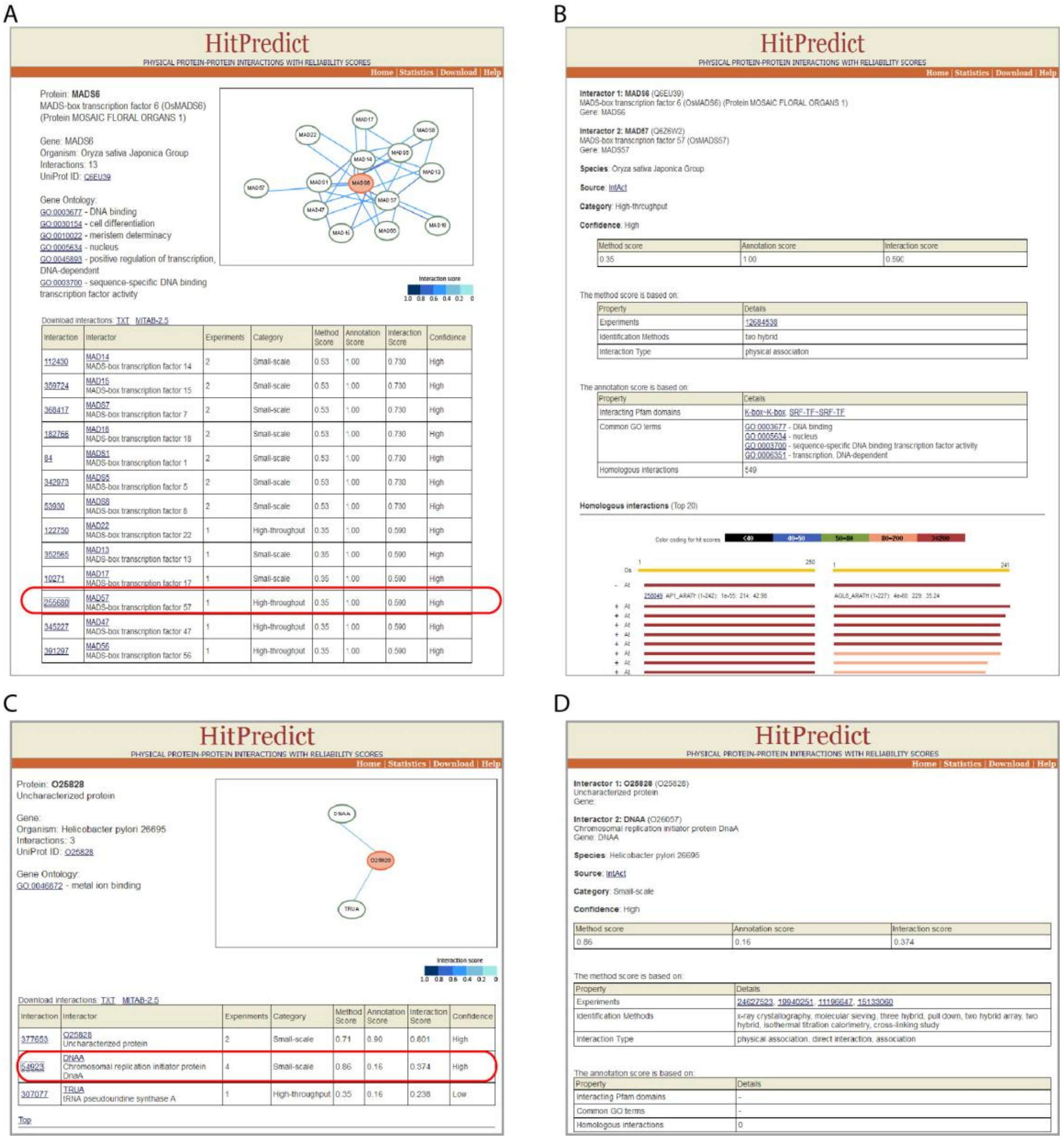

Supplement: Supplementary Data [file supp_bav117_Supplementary_information_r1.pdf]
